# Creation and pilot testing of cases for case-based learning: A pedagogical approach for pathology cancer diagnosis

**DOI:** 10.4102/ajlm.v6i1.637

**Published:** 2017-10-25

**Authors:** Shahin Sayed, Susan C. Lester, Michael Wilson, Daniel Berney, Ricard Masia, Zahir Moloo, Jennifer Stall, Alexia Eslan, Stephanie Ayers, Angela Mutuku, Jeannette Guarner

**Affiliations:** 1 Department of Pathology and Laboratory Medicine, Aga Khan University Hospital, Nairobi, Kenya; 2 Department of Pathology, Brigham and Women’s Hospital, Boston, Massachusetts, United States; 3 Department of Pathology, Harvard Medical School, Harvard University, Boston, Massachusetts, United States; 4 Department of Pathology and Laboratory Services, Denver Health, Denver, Colorado, United States; 5 University of Colorado School of Medicine, Aurora, Colorado, United States; 6 Barts Cancer Institute at Queen Mary University of London, London, United Kingdom; 7 Department of Pathology, Massachusetts General Hospital, Boston, Massachusetts, United States; 8 Department of Pathology, University of Ottawa, Ottawa, Ontario, Canada; 9 African Strategies for Advancing Pathology, Denver, Colorado, United States; 10 College of Pathologists of East, Central and Southern Africa, Nairobi, Kenya; 11 Department of Pathology and Laboratory Medicine, Emory University School of Medicine, Atlanta, Georgia, United States

## Abstract

**Background:**

Case-based learning (CBL) is an established pedagogical active learning method used in various disciplines and defined based on the field of study and type of case. The utility of CBL for teaching specific aspects of cancer diagnosis to practising pathologists has not been previously studied in sub-Saharan Africa.

**Objectives:**

We aimed to pilot test standardised cancer cases on a group of practising pathologists in sub-Saharan Africa to evaluate case content, clarity of questions and delivery of content.

**Methods:**

Expert faculty created cases for the four most commonly diagnosed cancers. The format included mini-cases and bullet cases which were all open-ended. The questions dealt with interpretation of clinical information, gross specimen examination, morphologic characteristics of tumours, ancillary testing, reporting and appropriate communication to clinicians.

**Results:**

Cases on breast, cervical, prostate and colorectal cancers were tested on seven practising pathologists. Each case took an average of 45–90 min to complete.

Questions that were particularly challenging to testers were on:

- Specimens they should have been but for some reason were not exposed to in routine practice.
- Ancillary testing and appropriate tumour staging.

New knowledge gained included tumour grading and assessment of radial margins. Revisions to cases were made based on testers’ feedback, which included rewording of questions to reduce ambiguity and adding of tables to clarify concepts.

**Conclusion:**

Cases were created for CBL in Kenya, but these are applicable elsewhere in Africa and beyond to teach cancer diagnosis. The pilot testing of cases prepared faculty for the actual CBL course and feedback provided by the testers assisted in improving the questions and impact on day-to-day practice.

## Introduction

The use of standardised cases to teach different levels of trainees in the medical professions is a common practice. The use of case-based learning (CBL), a well-established pedagogical active learning method with divergent definitions depending on the discipline and type of ‘case’ employed,^1^ to teach specific aspects of a cancer diagnosis to practising pathologists and pathology fellows or trainees is not frequently employed. Pathologists use daily sign-out cases on their desks to teach trainee residents. This approach has served many but is trainer dependent and based on the local practice rather than standard practice. As an example, if synoptic pathology reporting of cancers is not routine practice in a particular country, this will not be part of what is taught to resident trainees.

Case-based learning is a process by which trainees actively learn through a clinical presentation that serves as a stimulus to acquire additional knowledge on the specific clinical entity to solve problems. Williams^2^ emphasised that CBL is an educational paradigm posing contextualised questions that allow students to develop a collaborative approach to their education by fostering integrated learning and promoting self-assessment, reflection and life-long learning. In the medical setting, cases provide the student with the background of a patient or other clinical situation.^3^ The description can be vague but have adequate content to facilitate evaluation.^4,5^ In addition, supporting information that helps trainees acquire knowledge may include: vital signs, clinical signs and symptoms, laboratory results, book chapters and even the latest research articles. The instructor facilitates knowledge construction and directs students away from a predominantly passive, lecture-driven mode.^6^

Case-based learning is often contrasted with problem-based learning (PBL) and the differences between PBL and CBL are not always clear. Barrows and Tamblyn^7^ defined PBL as the learning that results from the process of working towards the understanding of a resolution of a problem where the problem is encountered first in the learning process.

Problem-based learning is more self-directed and allows students to explore various domains of the problem based on their prior knowledge but there is no guidance provided by facilitator even if learners deviate from the problem.^8,9^ In contrast, CBL is a guided inquiry method, with defined learning outcomes, and the teaching builds on prior knowledge, integrates data and considers application to future situations. This in turn encourages teamwork and accountability, as well as engages participants in their learning to think about plausible answers instead of passively receiving the information.^10^ Thus both CBL and PBL are used to stimulate and underpin the active acquisition of knowledge, skills and attitudes,^11^ although CBL is more structured.

This paper focuses on the process of creating standardised cancer cases and the pilot testing of the same. The pilot testing determined the validity of the questions and their acceptability. We describe the outcome and learning points from the pilot exercise and the improvements made on the cases based on the feedback received.

## Methods

### Ethical considerations

The Research Ethics Committee, Aga Khan University, approved this study (approval number: 2017/REC-39). A total of two senior and five mid-career faculty members provided written informed consent to participate in pilot testing of the cases in two sessions over an interval of three weeks. Testers were practising pathologists from a sub-Saharan Africa teaching institution with more than five years postgraduate teaching experience in surgical pathology.

### Study design

We elected to create cases that included the four most commonly diagnosed cancers by pathologists in the East, Central and Southern Africa regions: breast, cervical, prostate and colorectal cancers.^12,13^ The format of the cases included mini-cases (consisting of a narrative with tightly focused questions that assisted learners to apply a variety of concepts) and bullet cases (consisting of two to three sentences with one or two directed teaching points). All questions were open-ended so that the participants would write down an answer; there were no multiple-choice questions and only one correct answer. The questions in each of the cases were aimed at emphasising the pathological features of the cancers that have clinical impact on diagnosis, treatment and prognosis for the four selected cancers. The cases included questions that dealt with interpretation of clinical information (e.g. previous treatment, tumour marker data, other relevant history), aspects regarding description and processing of gross specimens (e.g. dissection and inking), morphologic characteristics of tumours (e.g. mitoses, grading, amount of tumour in biopsies), further ancillary testing (e.g. immunohistochemistry) and how tumours should be reported (including synoptic reporting) and the appropriate relevant communication to clinicians.

Answers to the questions were provided in PowerPoint format as reference material to the testers. The questions were formatted in a cascading style with an increasing level of complexity. The faculty created 10 mini cases (2 breast cases, 2 cervical cases, 3 colorectal cases and 3 prostate cases) in 14 to 50 questions and 4 bullet cases (all prostate) with 1–4 questions for each bullet case. After the cases were created, the expert faculty and facilitators reviewed and discussed the cases and questions prior to the pilot testing.

**Supplementary Figure 1:**
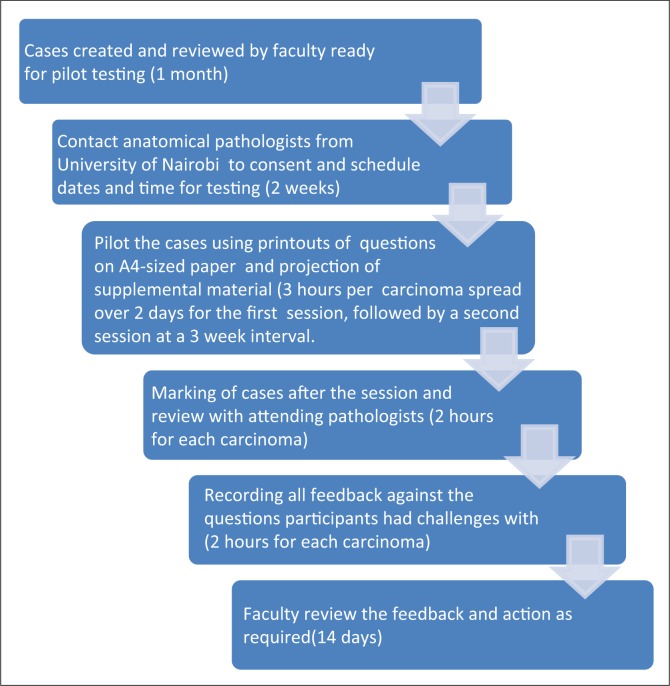
Flow chart of pilot testing process.

The aim of the testing was to evaluate case content and the clarity of the questions, and define the best manner of delivery (e.g. the best method to present microscopic images using glass slides and microscopes, printed photographs, or whole-slide digitised images on screens). We estimated the time each case would take to solve, so that testers would have an idea of the amount of uninterrupted time they would need to devote to testing of these cases. The process of creation of the cases and the pilot testing exercise is illustrated in Supplementary Figure 1.

### Pilot testing session

The main objectives of pilot testing the cases were to:

- Scrutinise the need for each question and document feedback from testing group participants.
- Review and improve upon the questions that were not clear.
- Determine the approximate length of time it took to complete each one of the cases.

A faculty member who had gone through the cases with the experts who created them facilitated the pilot testing session. Testers were practising pathologists from a Kenyan teaching institution with more than five years of postgraduate teaching experience in surgical pathology. The testers were given an overview of the capacity building project for which the cases were being created. They were informed about the objectives of the pilot testing and were asked to provide specific feedback regarding:

- Finding the answers, and the ease with which they found the answers, to the questions in the printed PowerPoint material provided.
- Information that was new to them after having solved the cases.
- The total time it took to answer each one of the cases and which questions took the most time to answer.

Other instructions given to testers included:

- They were encouraged to work in groups and advised to give themselves a block of 2–3 h to solve the cases for each cancer. However, they were asked to spend no more than 10 min per question on a case.
- They were told to go over the cases and answer the questions in each case in the sequence presented and to work without interruptions.
- They were provided with hard copies of the PowerPoint lectures and synoptic reporting templates and encouraged to refer to these so as to answer the questions.
- The participants were instructed to write down issues they saw with questions (e.g. responses could vary, could not find answer, too difficult, spent too much time).

Lastly, we (faculty and support staff) conducted a survey of testers to gain knowledge of acceptability of the standardised cases.

## Results

A total of two senior and five mid-career faculty members agreed to participate in pilot testing of the cases in two sessions over an interval of three weeks. Two cancers were pilot tested in each session. Table 1 provides details of the topics that were challenging to testers and the new concepts learned after the CBL exercise.

**TABLE 1:** Case type, challenges encountered and new knowledge gained.

Case type	Topic of challenging questions	New knowledge
Breast	Assessing for mitotic counts, grading of breast cancer, tumour-node-metastasis staging Interpretation of the Magee equation for estimating the 21-gene recurrence score Neo-adjuvant therapy and grossing of mastectomy/lumpectomy post neo-adjuvant therapy Interpretation of survival curves	Calculating field diameter of microscope Residual cancer burden score
Cervical	Histopathological evaluation of endo-cervical curettage Morphological features differentiating adenocarcinoma in situ from invasive adenocarcinoma Measurement of the depth of invasion of adenocarcinoma	Measurement of depth of invasion for adenocarcinoma Differentiating primary endo-cervical from endometrial cancer on immunohistochemistry
Colorectal	Staging of colorectal cancers Determination of radial margins molecular testing in colorectal cancers Grossing of rectal cancer resection specimens	Determination of appropriate radial margins Microsatellite instability testing in colorectal cancer Determination of completeness of meso-rectal resection
Prostate	Accurate identification of mimics of prostatic carcinoma Grade grouping was a challenge especially the concept of 4 + 3 = 7 (Grade group 3) not being categorised the same as 3 + 4 = 7 (Grade group 2) Determination of proportion and length of tumour in core biopsies Differentiating high-grade prostatic intraepithelial neoplasia from intra-ductal carcinoma	The new grade grouping system for prostate cancer High-grade prostatic intraepithelial neoplasia versus intra-ductal carcinoma – implications for management

Depending on the complexity of the case and the number of questions, each case took approximately 45 to 90 min to complete. The questions that were found to be challenging related to those cases and specimen types that the participants were not exposed to in routine practice within their local context. These included handling of breast specimens post neoadjuvant therapy, handling of rectal resection specimens and radical prostatectomy specimens. Other challenging questions included those related to molecular testing and use of complementary immunohistochemistry testing (e.g. microsatellite instability testing for colorectal cancer) and appropriate staging according to the tumour-node-metastasis system (e.g. determining whether serosal penetration is present in colorectal cancer). In addition, participants felt they had acquired new knowledge with regard to grading of tumours (assessing breast mitotic counts and the new grade group system for prostatic cancers), appropriately assessing radial margins for colorectal resection specimens and gained clarity on the indications and interpretation of the microsatellite instability testing for colorectal cancers. Based on the feedback given by the testers, changes were made to the cases. These changes included rewording of questions felt to be ambiguous or confusing and adding tables to some cases to clarify concepts (e.g. the distinction between tumour size and tumour stage).

**Supplementary Figure 2:**
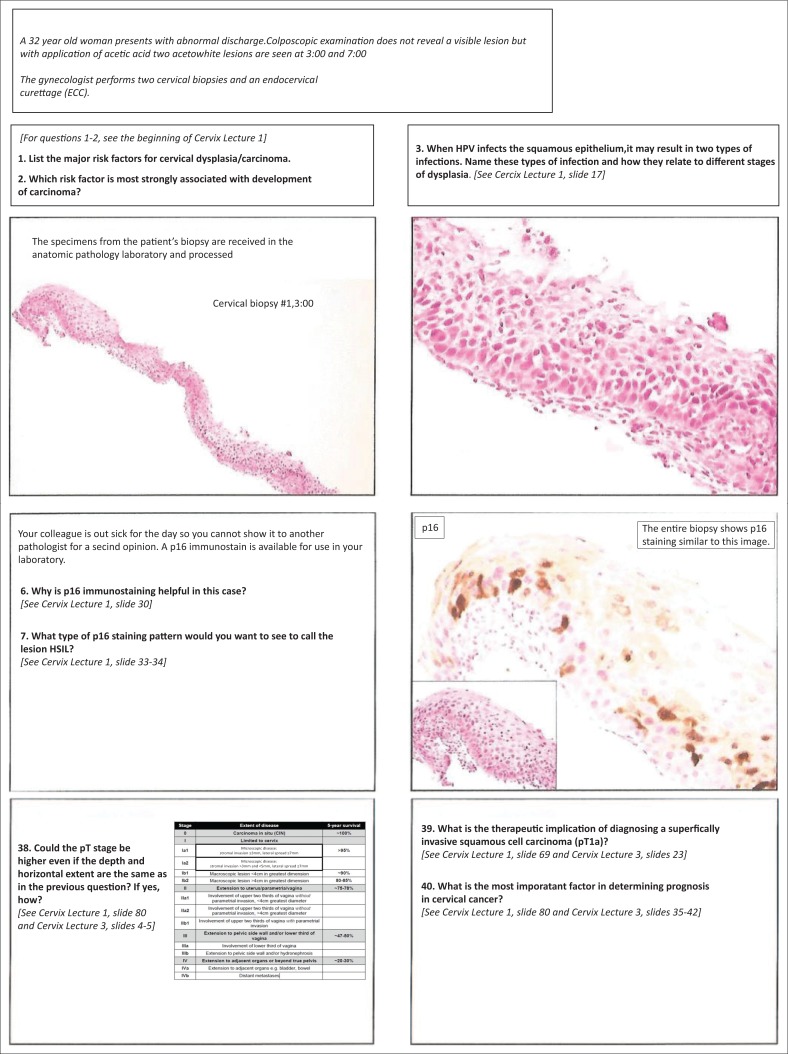
Examples of PowerPoint slides printed for pilot testing exercise.

Regarding the reference material given to the testers (i.e. the printed PowerPoint presentations), important feedback was provided. Participants noted that they should be instructed to review the presentations prior to attempting to answer the questions. In addition, participants suggested that it would be helpful to label the images in the presentations with the type of neoplasia or teaching point being demonstrated, such that the presentations could ‘stand alone’ as educational material (i.e. a live lecture was not necessary). There was debate among the expert faculty and facilitators regarding the presentation of the reference PowerPoint material to the testers (i.e. electronically rather than printed). The printed version on the A4-sized paper used for the testing had very small font size and was difficult to read (Supplementary Figure 2). Therefore, participants suggested that if printed material is used for the cases, the PowerPoint slides should be printed using a larger font size.

An electronic post-testing feedback survey was administered to the seven pilot testers three months after the exercise. Four of the testers who had taken part in the pilot testing exercise responded to the survey. They found the cases very useful and stated that the knowledge gained was applicable in their day-to-day practice. All of them commented that they have implemented changes in their practice based on what they learned from the CBL. Some of the testers, however, would have preferred video microscopy sessions with actual glass slides, longer duration of training sessions and prior access to reference or reading material in preparation for the cases.

## Discussion

The pilot testing of standard cancer cases provided information regarding acceptability, time it may take for pathologists in Kenya to solve these cases and knowledge gained. Moreover, at least one of the topics for each cancer in which the testers struggled were the same as the ones for which they gained knowledge. These cases were used one month later in a study that compared knowledge gained after a lecture-based course versus a CBL course for pathologists practising in East, Central and Southern Africa. The rationale of using the CBL type of instruction is that by ‘doing’ there is better retention of concepts with emphasis on critical thinking and comprehension of the defined problem and arriving at a solution in the appropriate context.^14,15^

In any residency training, review of cases with faculty is how residents learn. This system is faculty dependent and the content of instruction will vary from one faculty member to the next reviewing a case. In most instances, especially for pathology, the system provides opportunity for one-on-one teaching. The training content in this context is subject to the local practice. In addition, if a case of a rare entity is unavailable during the trainee’s residency years, the resident misses out on the opportunity to see such a case, thus limiting their exposure and learning. Creating cases for CBL therefore allows for standardised instruction to be provided across the residency years.

Testers of our cases felt that the material was easy to relate to and that the amount of content and the complexity included was appropriate to their needs and daily practice. We included mini cases and bullet cases, but the two different format types and lengths of questions did not make a difference to the participants’ level of engagement. According to Milliard et al.,^16^ the success of a case is highly dependent upon the interest it elicits among the students in the synthesis and applicability of the information provided for the learning process.^16^ In order to create student engagement and promote in-depth discussions, case length, realism and level of intrigue should be considered.^17,18^

Our cases had all the essential facts to facilitate integration of the information provided so as to arrive at a predetermined definitive solution. We did not have questions that allowed multiple possible solutions.^19^ The answers to the questions were present in the PowerPoint presentations that were given to the testers at the same time they were given the cases, but the majority of the testers found it distracting to refer to the materials. This could be explained by the fact that the PowerPoint presentations were printed with two slides per page using letter-size paper. This meant that the font size was very small and may have discouraged participants from utilising the materials. Faculty therefore agreed that when using these cases an electronic version of the reference material needs to be provided or the presentations should be printed as one slide per page.

We established a set of guidelines for the testers which they were required to adhere to when solving the cases. Two factors may be key to the success of a CBL platform: group activation and accompanying peer instruction.^15,20,21^ As an example, it may be useful to illustrate the benefits of teamwork^15^ or in our cases the need to answer the questions in order as each built upon the previous answer. Introducing CBL pedagogies to students for the first time may be bit of a challenge as it may result in resistance; therefore, one should take into consideration that the instructor must be skilled in facilitating group learning activities. Clear instructions on the CBL template, goals and timelines and provision of the case prior to the learning activity can mitigate this challenge.^15,22^ Failure of CBL implementation is mainly caused by poor instructional planning as previously reported by Struyven et al.^23^

Our seven testers worked together to solve the cases. Some have stated that six participants in a group should be the maximum group size permitted as it allows for closer interaction and participation of each group member.^22^ The discussion should be paced to ensure that trainees and instructors alike become familiarised with the CBL style.^15^ As cited by Kulak and Newton,^15^ in order to strengthen instructor–student engagement, the facilitator should familiarise themselves with student names. The amount of time that it took for questions to be answered by the testers was between 45 and 90, minutes depending upon the case. This was very similar to what was expected by the faculty, but testing the cases was nonetheless instrumental in confirming the amount of time required for case completion in this particular context. After the testing, the faculty decided to include references in the cases to guide participants to the location of the pertinent information in the presentations, as it was deemed to be too time-consuming to locate the information otherwise. To optimise time utilisation and sustain a focused discussion on the case in hand, the instructor ideally should be familiar with all aspects of the case.^15,24^

We found that very minor changes were needed for the cases and questions; however, the expert faculty that created the cases found the feedback from the testers very informative. It allowed the expert faculty who practise in a Western context to become familiar with African realities and tailor the instructional delivery of the cases accordingly. Having a faculty familiar with the local environment to conduct the pilot testing also permitted the group members a level of comfort in expressing their views, providing honest feedback and remaining engaged throughout the process.

In summary, we created standardised cancer cases for CBL in East, Central and Southern Africa, but these same cases could be used elsewhere to teach cancer diagnosis. Our pilot testing prepared us better for the actual CBL course and gave us a sense of possible problems to encounter. In the survey performed three months later, testers stated that they had changed their practice to incorporate the concepts learned. As an example, testers indicated that they had adopted the new prostate grading system into practice. This emphasises that this active pedagogical approach to learning pathological concepts for cancer diagnosis was beneficial to our testers who seemed to have learned as much as if they had participated in the course.
